# Autologous Tooth-Derived Biomaterials in Alveolar Bone Regeneration: A Systematic Review of Clinical Outcomes and Histological Evidence

**DOI:** 10.3390/jfb16100367

**Published:** 2025-10-01

**Authors:** Angelo Michele Inchingolo, Grazia Marinelli, Francesco Inchingolo, Roberto Vito Giorgio, Valeria Colonna, Benito Francesco Pio Pennacchio, Massimo Del Fabbro, Gianluca Tartaglia, Andrea Palermo, Alessio Danilo Inchingolo, Gianna Dipalma

**Affiliations:** 1 Department of Interdisciplinary Medicine, University of Bari “Aldo Moro”, 70121 Bari, Italy; angelomichele.inchingolo@uniba.it (A.M.I.); graziamarinelli@live.it (G.M.); robertovito.giorgio@uniba.it (R.V.G.); valeriacolonna91@gmail.com (V.C.); benitofrancescopio.pennacchio@uniba.it (B.F.P.P.); alessiodanilo.inchingolo@uniba.it (A.D.I.); gianna.dipalma@uniba.it (G.D.); 2 Department of Biomedical, Surgical and Dental Sciences, Milano University, 20122 Milan, Italygianluca.tartaglia@unimi.it (G.T.); 3 Fondazione IRCCS Ca’ Granda Ospedale Maggiore Policlinico, Unit of Maxillo-FacialSurgery and Dentistry, 20122 Milan, Italy; 4 Department of Experimental Medicine, University of Salento, 73100 Lecce, Italy; andrea.palermo@unisalento.it

**Keywords:** autologous dentin graft, alveolar bone regeneration, demineralized dentin matrix, bone graft substitute, histomorphometric analysis, cone beam computed tomography, dental biomaterials, tissue regeneration

## Abstract

Background: Autologous tooth-derived grafts have recently gained attention as an innovative alternative to conventional biomaterials for alveolar ridge preservation (ARP) and augmentation (ARA). Their structural similarity to bone and osteoinductive potential support clinical use. Methods: This systematic review was conducted according to PRISMA 2020 guidelines and registered in PROSPERO (CRD420251108128). A comprehensive search was performed in PubMed, Scopus, and Web of Science (2010–2025). Randomized controlled trials (RCTs), split-mouth, and prospective clinical studies evaluating autologous dentin-derived grafts were included. Two reviewers independently extracted data and assessed risk of bias using Cochrane RoB 2.0 (for RCTs) and ROBINS-I (for non-randomized studies). Results: Nine studies involving 321 patients were included. Autologous dentin grafts effectively preserved ridge dimensions, with horizontal and vertical bone loss significantly reduced compared to controls. Histomorphometric analyses reported 42–56% new bone formation within 4–6 months, with minimal residual graft particles and favorable vascularization. Implant survival ranged from 96–100%, with stable marginal bone levels and no major complications. Conclusions: Autologous tooth-derived biomaterials represent a safe, biologically active, and cost-effective option for alveolar bone regeneration, showing comparable or superior results to xenografts and autologous bone. Further standardized, long-term RCTs are warranted to confirm their role in clinical practice.

## 1. Introduction

Maintaining adequate alveolar ridge volume after tooth extraction is critical for achieving both functional and aesthetic success in implant dentistry. Post-extraction socket remodeling is a well-documented biological process that leads to progressive horizontal and vertical bone resorption. These dimensional changes can complicate implant placement, especially in the anterior maxilla or in patients with limited residual bone, thus requiring additional regenerative interventions [1,2].

To prevent or compensate for such resorption, various bone grafting materials have been introduced in clinical practice. These include autologous bone, xenografts (e.g., deproteinized bovine bone), allografts, and alloplastic substitutes. While widely used, each of these graft types has inherent limitations [2,3,4]. Autologous bone, although considered the gold standard due to its osteogenic potential, requires harvesting from a donor site, increasing surgical morbidity. Xenografts and allografts carry potential immunogenic or ethical concerns, and synthetic biomaterials often lack biological activity [5,6,7].

In this context, tooth-derived autologous biomaterials have emerged as an innovative and biologically compatible option for alveolar bone regeneration (ABR). The foundation of this approach lies in the anatomical and biochemical similarity between dentin and bone [8,9,10]. Dentin is composed of approximately 70% hydroxyapatite (HA), 20% organic matrix (primarily type I collagen), and 10% water, closely resembling the composition of cortical bone. Moreover, dentin contains bioactive molecules such as bone morphogenetic proteins (BMPs), transforming growth factor beta (TGF-β), and dentin matrix protein 1 (DMP-1), which contribute to osteoinductive activity [11,12].

**Figure 1:**
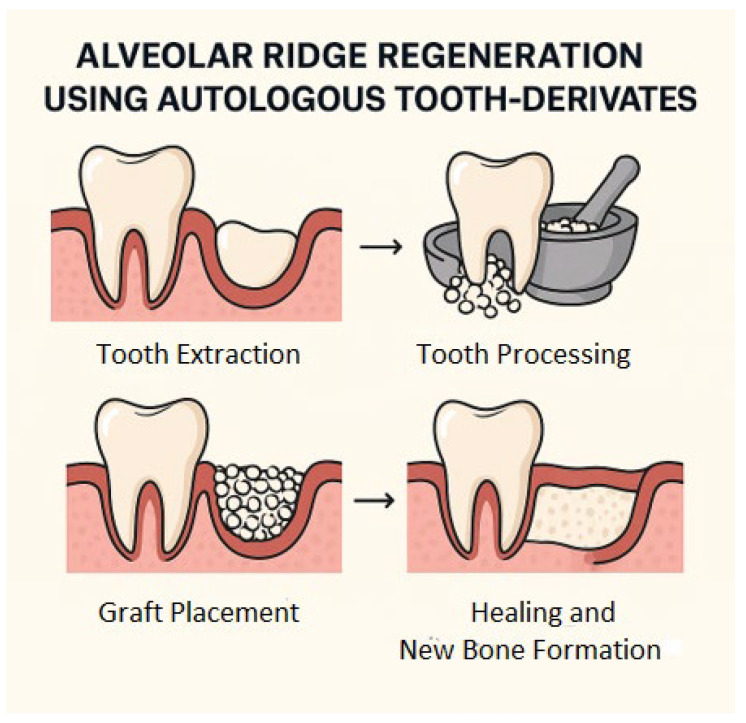
Schematic representation of the clinical workflow for autologous tooth-derived biomaterials in alveolar bone regeneration: tooth extraction, biomaterial processing, graft placement, and bone healing.

The transformation of extracted teeth into usable grafting material is facilitated by various processing methods, including demineralized dentin matrix (DDM) preparation, particulate grinding, or the creation of dentin blocks. These procedures aim to expose the bioactive proteins embedded within the dentin and to enhance the material’s osteoconductive and osteoinductive capabilities [11,12]. The result is a graft material that is not only structurally suitable for bone regeneration but also biologically active—capable of stimulating new bone formation without the risk of immune rejection or cross-contamination [13,14,15,16] (Figure 1).

The clinical use of autologous tooth-derived grafts (ATDGs) is now increasingly feasible due to the availability of chairside processing systems, which allow immediate preparation of sterile graft material during the same surgical session in which tooth extraction occurs [11,12,17]. This real-time conversion of extracted teeth into a usable bone substitute supports the principles of minimally invasive surgery, sustainability, and personalized medicine. It also reduces overall treatment costs and eliminates the need for donor site surgeries, which are associated with increased pain, bleeding, and healing time [18,19,20].

In alveolar ridge preservation (ARP), ATDGs have shown promising results in maintaining the volume and contour of the extraction site. Clinical measurements and radiographic evaluations using cone beam computed tomography (CBCT) indicate limited resorption and favorable dimensional stability when these materials are used [21,22]. Similarly, in ridge augmentation procedures—both horizontal and vertical—dentin blocks and particulated grafts have demonstrated effective space maintenance and integration with the host bone [23,24,25,26].

Histological evaluations provide further support for the efficacy of these biomaterials. Studies report formation of new vital bone interspersed with residual dentin particles, with evidence of early vascularization and minimal inflammatory response. Dentin granules appear to act as a scaffold for osteoprogenitor cell migration and matrix deposition [27,28,29]. Over time, these grafts are gradually resorbed and replaced by newly formed bone, often exhibiting characteristics of mature lamellar structure. The osteoconductive and bioactive nature of the material is evident from both microscopic and immunohistochemical analyses [30,31,32].

Another notable advantage of ATDGs is their favorable handling properties during surgery. The material conforms well to the defect geometry and can be easily combined with other regenerative tools, such as collagen membranes or platelet-rich fibrin (PRF), to enhance healing [33,34,35]. Their mechanical rigidity, especially when used in block form, also allows for stability in space-critical defects where structural support is essential.

Despite these benefits, challenges remain in standardizing protocols for processing, sterilization, and particle size optimization [36,37,38,39]. Variations in the source material—such as the type of tooth, previous endodontic treatment, or presence of caries—may affect the graft’s regenerative performance. In addition, while short- and medium-term results appear favorable, long-term data on implant survival rates, bone stability, and functional outcomes are still limited [40,41,42].

To address these gaps, it is essential to systematically review the existing clinical literature that evaluates the regenerative performance of ATDGs in human subjects [43,44,45]. This review focuses on high-quality clinical studies, including randomized controlled trials (RCTs), controlled prospective studies, and split-mouth designs, which assess the efficacy of autologous dentin-based grafts for ARP and alveolar ridge augmentation (ARA) [21,22,46]. The review also examines outcomes from both clinical and histological perspectives, such as the following:

Primary outcomes: new bone formation (assessed histomorphometrically), ridge dimensional stability (measured by CBCT or conventional radiography).

Secondary outcomes: percentage of residual graft material, rate of bone resorption, presence of complications, and histologic integration quality.

Furthermore, the included studies compare ATDGs with other commonly used graft materials, such as xenografts (e.g., Bio-Oss^®^), autologous bone, PRF, and untreated extraction sites, allowing for a meaningful analysis of comparative performance [47,48,49].

This systematic review aims to provide a comprehensive and evidence-based evaluation of tooth-derived grafts in ABR, offering valuable insights for clinicians, researchers, and biomaterial developers [50]. As regenerative dentistry continues to evolve toward more biologically informed and patient-specific solutions, the repurposing of extracted teeth into functional grafting materials could represent a paradigm shift in clinical practice [51,52,53]. By synthesizing the available clinical and histologic evidence, this work intends to clarify the potential, safety, and limitations of ATDGs as a viable alternative in modern bone regeneration strategies [54,55,56].

## 2. Materials and Methods

The increasing clinical relevance of autologous tooth-derived biomaterials in oral surgery has led to growing interest in their regenerative potential. This systematic review aims to compare autologous dentin-based grafts with other commonly used bone substitutes in terms of clinical and histological outcomes in ABR.

By providing a structured, evidence-based analysis, this study seeks to guide oral surgeons and clinicians in evaluating the performance and limitations of tooth-derived grafts compared to xenografts, autografts, and alloplastic materials.

### 2.1. Search Processing

A comprehensive literature search was performed using the electronic databases PubMed, Scopus, and Web of Science. The search period was limited from January 2010 to May 2025. The search terms included combinations of the following keywords using Boolean operators: (“ATDG” OR “dentin graft” OR “autogenous dentin” OR “tooth graft”) AND (“ARP” OR “socket preservation” OR “bone regeneration”) AND (“clinical study” OR “histological analysis” OR “randomized controlled trial”).

Protocol and Registration:

This systematic review was conducted in accordance with the PRISMA 2020 guidelines and registered in PROSPERO (Registration No. CRD420251108128).

### 2.2. Inclusion and Exclusion Criteria

Searches were restricted to English-language articles published between 2015 and 2025 and conducted on human subjects. Only clinical studies using autologous dentin or tooth-derived materials for ARP or augmentation were considered.

Studies were selected based on the following inclusion criteria:

Human clinical studies (RCTs, cohort, and case–control studies); use of ATDG(s) for bone regeneration procedures; comparative or observational outcomes regarding clinical, radiographic, or histologic endpoints; minimum follow-up duration of 3 months; published in English.

The inclusion of randomized controlled trials (RCTs), split-mouth studies, and prospective clinical trials was justified by their ability to minimize bias, reduce inter-individual variability, and provide longitudinal clinical and histological data on this emerging class of biomaterials.

Exclusion criteria were the following:

In vitro or animal studies; case reports, letters, or reviews; studies involving non-autologous materials only; lack of quantitative outcome data.

### 2.3. PICO Question

The PICO question (Table 1) addressed was:

### 2.4. Data Processing

Data extraction was conducted using a pre-piloted spreadsheet in Excel, used by two independent reviewers. Extracted information included author names, publication year, study design, sample size and characteristics, type of graft material used, surgical application (e.g., socket preservation, ridge augmentation), comparator groups, outcome measures, and follow-up duration. The ROBINS-I tool for non-randomized studies and the Cochrane RoB 2.0 tool for RCTs were used to evaluate the risk of bias. Any disagreements between the two reviewers were resolved through discussion and, when necessary, with input from a third reviewer. This process ensured the reliability and consistency of the data included in the final synthesis. Disagreements were resolved through discussion or consultation with a third reviewer.

To improve reproducibility, details regarding donor tooth type, graft preparation (e.g., degree of demineralization, particle vs. block form), sterilization/disinfection method (autoclave, chemical disinfection, proprietary systems), and characterization techniques (histomorphometry, CBCT, immunohistochemistry) were systematically extracted and summarized in a Supplementary Table S1.

### 2.5. Grouping of Studies for Synthesis

The included studies were grouped according to: (i) type of autologous tooth-derived biomaterial (particulate dentin, demineralized dentin matrix, dentin block), and (ii) clinical application (alveolar ridge preservation, alveolar ridge augmentation, or guided bone regeneration).

### 2.6. Effect Measures

Effect measures included mean dimensional changes of alveolar ridge width and height (in millimeters), percentage of new bone formation and residual graft (histomorphometry), implant survival rates (%), and occurrence of complications.

### 2.7. Synthesis Methods

Due to heterogeneity in study design and outcome measures, a narrative synthesis was performed. Quantitative data (means, standard deviations, and percentages) were extracted and presented in comparative tables. Where possible, ranges of outcomes across studies were summarized.

### 2.8. Certainty Assessment

The certainty of evidence was qualitatively assessed by considering study design, sample size, follow-up duration, and risk of bias evaluations. Although a formal GRADE analysis was not feasible, limitations in methodology, variability of protocols, and short follow-up periods were explicitly highlighted in the discussion.

## 3. Results

### 3.1. Study Selection and Characteristics

**Figure 2:**
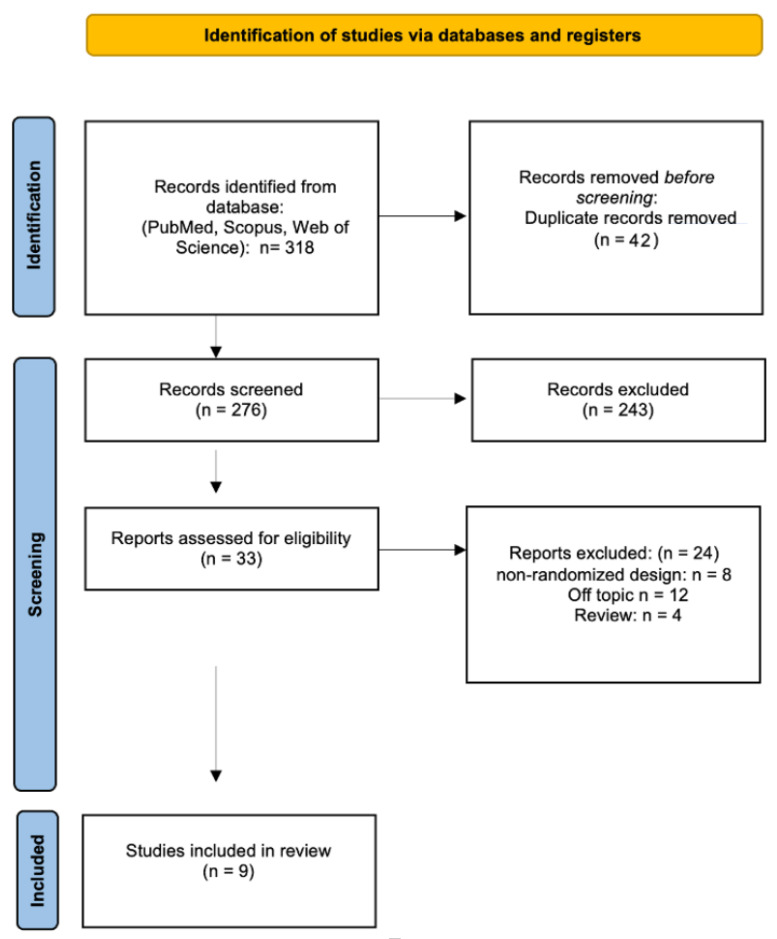
PRISMA flowchart. This diagram summarizes the screening and selection process of eligible studies, ensuring transparency and methodological rigor [58].

The initial database search, conducted according to PRISMA guidelines, retrieved a total of 318 records. After removing duplicates, 276 articles were screened by title and abstract. Of these, 243 were excluded, and 33 full-text articles were assessed for eligibility. 24 studies were excluded for reasons such as non-randomized design, irrelevant outcomes, or ineligible patient populations. Finally, 9 studies met all inclusion criteria and were included in the qualitative synthesis. The selection process is illustrated in the PRISMA 2020 flowchart (Figure 2).

### 3.2. Study Characteristics

The included studies were published between 2015 and 2025 and involved a total of 321 patients undergoing various bone regeneration procedures using autologous tooth-derived materials. Interventions included (DDM), mineralized dentin particulate (MDP), and dentin blocks (DB), which were compared with xenografts (e.g., Bio-Oss), autologous bone, collagen membranes, platelet-rich fibrin (PRF), or blood clot, and in some cases, with no graft material. Clinical outcome assessed included primary outcomes such as new bone formation (evaluated via histomorphometry) and ridge dimensional changes (measured through CBCT), as well as secondary outcomes including residual graft presence, complications, material integration, and implant placement success.

The follow-up durations across studies ranged from 3 to 24 months. Study designs included RCTs, split-mouth studies, and prospective clinical trials. Sample sizes, patient demographics, interventions, comparators, and outcome measures were systematically extracted.

Data extraction was independently performed by two authors (A.M.I. and A.D.I.) using a predefined collection form. Extracted variables are summarized in Table 2. Any discrepancies during data extraction were resolved by consensus or, when necessary, adjudicated by a third reviewer (G.M.).

A total of nine studies were included, consisting of six randomized controlled trials, one split-mouth study, and two prospective studies. The publications span from 2015 to 2024: one study in 2015, one in 2017, one in 2018, one in 2020, two in 2021, two in 2022, and one in 2024. The trend shows an increase in the last five years, with more than half of the studies published from 2020 onwards.

### 3.3. Clinical and Radiographic Outcomes

Across studies, autologous dentin grafts demonstrated effective preservation of alveolar ridge dimensions, such as ridge width (horizontal dimension) and ridge height (vertical dimension), with ridge width reductions ranging from 0.9 to 2.5 mm at 6 months. In split-mouth designs, sites treated with dentin-derived grafts showed significantly less vertical and horizontal bone loss compared to ungrafted controls. Volumetric analyses confirmed a superior preservation effect relative to xenografts and spontaneous healing.

### 3.4. Histological Outcomes

Histomorphometric analyses revealed new bone formation ranging from 42% [59] to 56% (canat 4 to 6 months post-surgery, with residual graft particles being minimal (7–11%) (Radoczy-Drajko et al., 2021) [60]). Histological sections consistently showed integration of dentin particles with surrounding bone, active osteogenesis, and absence of inflammatory infiltrates (López Sacristán et al., 2024; Hussain et al., 2023 [61,62]). Immunohistochemical studies in some trials confirmed the presence of osteogenic markers and low proinflammatory cytokine expression in grafted areas Ouyang et al. (2024) [63].

### 3.5. Implant Outcomes

Implants placed in sites previously augmented with autologous dentin grafts exhibited high survival rates (96.4% in Pohl et al., 2017 [64]; 95.6% in Li et al., 2018 [65]) and stable marginal bone levels at 12–24 months (Pohl et al., 2017 [64]; Ouyang et al., 2024 [63]). Primary stability and osseointegration were consistently reported as comparable to those achieved with conventional graft materials (Elraee et al., 2022 [59]; Li et al., 2018 [65]).

### 3.6. Complications

Postoperative complications were rare and minor across all studies. No cases of graft rejection, infection, or adverse inflammatory response were reported. Patient tolerance and intraoperative handling were rated favorably.

Overall, the evidence supports the clinical effectiveness and biological compatibility of ATDG(s) for ABR in various contexts.

**Table 2:** Summary of selected studies.

Author (Year)	Design	Country	Purpose	Sample Size (I/C)	Mean Age (I/C)	Population	Intervention (I)	Comparator (C)	Outcome (O)	Follow-Up
Elfana et al. (2021) [66]	RCT	Egypt	Evaluate efficacy of whole-tooth vs demineralized dentin graft in ARP	20/20	42.6 ± 6.2/41.9 ± 5.8	Patients undergoing tooth extraction	Autogenous whole-tooth graft for ARP	Demineralized dentin graft	Dimensional changes of the alveolar ridge, radiographic and clinical evaluation	At 6 months: ridge width loss 1.2 mm vs. 2.4 mm (*p* < 0.05). New bone: 48.9% vs. 21.5% (*p* < 0.001). Conclusion: demineralized dentin is superior.
Elraee et al. (2022) [59]	RCT	Egypt	Compare dentin vs autogenous bone block for ridge augmentation	18/18	41.2 ± 6.9/42.1 ± 7.0	Patients requiring horizontal ridge augmentation	Autogenous dentin block graft for ridge augmentation	Autogenous bone block graft	Clinical and histological evaluation of horizontal ridge width gain, bone quality, and implant feasibility	At 6 months: horizontal gain 3.52 mm vs. 2.24 mm (*p* < 0.05). Histology: 42% new bone both groups, residual dentin 30%. Conclusion: dentin maintains volume longer.
Hussain et al. (2023) [62]	RCT	Iraq	Evaluate autogenous dentin biomaterial in socket preservation	29/29	39.8 ± 8.2/40.1 ± 7.9	Patients undergoing single tooth extraction	Autogenous dentin graft	Natural healing (no graft)	Radiographic evaluation of bone height and width preservation	At 3 months: horizontal bone loss 0.9 mm vs. 2.5 mm (*p* < 0.01). Histology: vital bone, minimal remnants. Conclusion: ADB is safe and effective.
Li et al. (2018) [65]	RCT	China	Compare DDM vs Bio-Oss in GBR with immediate implants	20/20	40.6 ± 5.8/41.1 ± 6.1	Patients requiring immediate implant placement in periodontal post-extraction sites	Autogenous DDM in GBR	Bio-Oss granules in GBR	Radiographic assessment of bone volume around implants	At 6 months: implant ISQ stability similar; new bone 45% vs. 38% (NS). Implant survival 95.6%. Conclusion: DDM is comparable to xenograft.
López Sacristán et al. (2024) [61]	Split-mouth clinical study	Spain	Compare ATDG with spontaneous healing in bilateral sockets	15/15	46.2 ± 7.5/45.8 ± 7.1	Patients requiring bilateral tooth extraction	ATDG in post-extraction socket	Contralateral socket left to heal naturally	Radiological and histological analysis of bone regeneration and socket preservation	At 4 months: ridge width loss 12.8% vs. 26% (*p* < 0.05). Histology: osteogenesis and integration. Conclusion: ATDG is effective.
Oguić et al. (2023) [67]	RCT	Croatia	Assess autologous dentin graft vs bovine/autologous mix in esthetic zone	22/22	44.9 ± 7.4/45.6 ± 7.3	Patients requiring grafting in the esthetic zone	Autologous dentin graft	Bovine xenograft mixed with autologous bone	Radiographic, histological, and immunohistochemical evaluation of osteogenesis and graft integration	At 6 months: bone fill is similar. Immunohistochemistry: lower TNF-α, BMP-4 in dentin group. Conclusion: favorable remodeling, reduced inflammation
Ouyang et al. (2024) [63]	RCT	China	Compare APDDM vs DBBM in orthodontic patients with alveolar deficiency	30/30	37.9 ± 9.1/38.4 ± 8.8	Orthodontic patients with alveolar bone deficiency (n = 60)	Partially demineralized dentin matrix(APDDM)	Deproteinized bovine bone mineral (DBBM)	Dimensional gain in bone width and height, reduced postoperative pain and swelling, similar long-term bone stability	At 6 months: gain width 3.5 ± 0.8 mm vs. 2.2 ± 0.6 mm (*p* = 0.002). At 24 months: resorption higher in APDDM. Conclusion: faster turnover, less discomfort.
Pohl (2017) [64]	Open prospective clinical study	Austria	Evaluate untreated tooth grafts for ridge augmentation	20	49.7 ± 10.2	Patients requiring lateral ridge augmentation or intraosseous defect filling (n = 20)	Autogenous unaltered tooth material (blocks and particulate)	No direct comparator; descriptive clinical cohort	Implant survival rate (96.4%), peri-implant bone loss (0.58 mm at 2 years), probing depth (1.7 mm), no inflammation	At 24 months: implant survival 96.4%; marginal bone loss 0.58 mm. Conclusion: tooth graft is effective, osteoconductive.
Radoczy-Drajko et al. (2021) [60]	Prospective clinical study	Hungary	Assess autologous tooth particulate graft in ridge preservation	25/25	51.3 ± 7.1/50.9 ± 6.8	Patients with extraction defect score class 3–4 post-extraction defects	Autogenous tooth-derived particulate graft for ridge preservation	No graft (natural healing)	Clinical, radiographic and histological evaluation of bone regeneration and ridge preservation	At 6 months: horizontal reduction 15% vs 26% (*p* < 0.05). New bone 56% vs. 42%. Conclusion: graft improved preservation

### 3.7. Risk of Bias Assessment

The risk of bias was evaluated using the following:

Three reviewers (V.C., R.V.G., and B.F.P.P.) assessed each study across the following domains of risk of bias:

#### 3.7.1. ROBINS-I Domains (Non-Randomized Studies) (Table 3)

- Bias due to confounding
- Bias in the selection of participants
- Bias in the classification of interventions
- Bias due to deviations from intended interventions
- Bias due to missing data
- Bias in the measurement of outcomes
- Bias in the selection of the reported result

#### 3.7.2. RoB-2 Domains (Randomized Studies) (Table 4)

- Bias arising from the randomization process
- Bias due to deviations from intended interventions
- Bias due to missing outcome data
- Bias in the measurement of the outcome
- Bias in the selection of the reported result

Each study was rated as having low, moderate, or high risk of bias, based on the highest risk domain identified.

- Low-risk studies employed appropriate randomization, maintained blinding when applicable, and reported outcomes completely and transparently.
- Moderate risk studies lacked blinding or had some methodological limitations but retained informative value.
- High-risk studies showed clear issues with randomization, management of deviations, or incomplete outcome data, limiting the internal validity of results.

Elfana et al. (2021) [66]—Low Risk: This RCT demonstrated an appropriate study design with clearly defined inclusion criteria, adequate randomization, and outcome assessors. However, the lack of blinding may have introduced minor detection bias, although the overall methodology supports a low-risk classification.

Radoczy-Drajko et al. (2021) [60]—Moderate Risk: As a prospective non-randomized clinical study without blinding, this study was prone to bias due to participant selection and possible confounding variables. While clinical and histological outcomes were clearly reported, the absence of a control group with standard treatment and the small sample size increase susceptibility to bias.

López Sacristán et al. (2024) [61]—Moderate Risk: Although designed as a split-mouth clinical study (which minimizes intersubject variability), the study lacked random allocation and blinding. The clear reporting of histological and radiological outcomes supports moderate-risk classification, though the absence of operator blinding and subjective outcomes affects internal validity.

Hussain et al. (2023) [62]—Low Risk: This RCT was conducted with a solid methodology, including proper randomization and defined clinical endpoints. Although patient blinding was not feasible, objective radiographic measures and consistent follow-up reduced detection and performance bias.

Ouyang et al. (2024) [63]—Low Risk: This RCT featured a robust sample size, standardized intervention protocols, and a long-term follow-up (24 months). The use of validated outcome measures and appropriate comparator DBBM supports its classification as low risk.

Elraee et al. (2022) [59]—Low Risk: This study provided well-described randomization procedures, clear intervention comparisons, and histological validation. Despite a short follow-up period (6 months), the design and reporting were rigorous, placing it in the low-risk category.

Li et al. (2018) [65]—Low Risk: Randomization, defined inclusion criteria, and comprehensive outcome analysis (including implant stability and bone volume) reduce the possibility of significant bias. Blinding of outcome assessors further supports this classification.

Oguić et al. (2023) [67]—Low Risk: Despite the complexity of histological and immunohistochemical analysis, the study followed a randomized controlled design with robust control (bovine-autologous mix). The presence of objective data, blinding, and detailed methodology justified the low-bias rating.

Pohl et al. (2017) [64]—Moderate Risk: This open prospective study lacked randomization and a direct comparator, increasing the potential for selection bias and limiting internal validity. However, the clear reporting of clinical outcomes, implant success rates, and follow-up at 24 months supports a moderate-risk classification rather than a high one, particularly given the observational nature and practical relevance of the results.

## 4. Discussion

Autologous tooth-derived grafts (ATDGs) are emerging as a biologically active alternative to conventional materials for ABR, supported by growing clinical and histological evidence in the context of oral and implant surgery. This systematic review includes nine clinical studies conducted between 2015 and 2025, which evaluate the effectiveness of various dentin-derived biomaterials—such as particulates, demineralized dentin, and dentin blocks—in ARP and ARA, in comparison with xenografts, autologous bone, or untreated sites [68,69,70].

Among the first clinical studies to directly compare two types of tooth-derived grafts, Elfana et al. evaluated the efficacy of autogenous whole-tooth graft and autogenous demineralized dentin graft (ADDG) in ridge preservation [66]. After six months, both materials significantly mitigated dimensional bone loss compared to historical data on ungrafted sites [66]. ADDG, in particular, promoted greater new bone formation and lower residual graft percentage—an effect attributed to increased exposure of growth factors such as TGF-β, IGF-II, and BMP-2 released through dentin demineralization. Collagen fiber exposure also facilitated material remodeling [71,72,73]. However, the study excluded molar regions and did not assess patient-reported outcomes, such as postoperative pain.

Supporting the regenerative potential of dentin-derived materials, Radoczy-Drajko et al. conducted a pilot study on Bonmaker^®^, a graft derived from mechanically and chemically processed autologous teeth [60]. After six months, a 15% mean reduction in horizontal volume was observed, along with an 18.3% gain in vertical height. Histological analysis showed 56% new bone formation and only 7% residual particles, with evidence of active osteogenesis—confirmed by the presence of osteoblasts, osteoid tissue, and resorption lacunae containing multinucleated osteoclasts [73,74,75]. The material also demonstrated excellent handling and adherence when hydrated, facilitating adaptation to the recipient site. Implant integration was optimal, with stable implants placed in vital bone, and ridge preservation was maintained after 12 months.

In a split-mouth study by López Sacristán et al., fresh autologous dentin grafts were compared to spontaneous healing post-extraction [61]. The treated sites exhibited a 12.8% reduction in ridge width, compared to 26% in controls [76,77,78,79]. Histological analysis confirmed integration of dentin particles within the newly formed tissue without inflammatory response, suggesting osteoinductive potential despite the absence of chemical treatment [80,81].

Another significant contribution came from Hussainet al., who evaluated the use of autogenous dentin biomaterial (ADB) in 29 patients undergoing non-molar tooth extractions [62]. The treated group showed superior preservation of bone dimensions and more mature trabecular regeneration, with minimal material remnants and no signs of inflammation [62]. Compared to xenografts reported in the literature, ADB demonstrated faster remodeling and fewer complications, making it a safe and cost-effective option [82,83,84].

In the context of immediate post-extraction guided bone regeneration (GBR) in patients with advanced periodontitis, Li et al. [65] showed that (DDM) fully integrated into host bone within six months, achieving implant stability (ISQ) comparable to the Bio-Oss^®^ control group. The absence of significant differences in marginal bone loss and the 95.6% implant success rate support the use of this autologous resource even in challenging clinical scenarios.

Histological and immunohistochemical analyses conducted by Oguićet al. compared autologous demineralized dentin graft (ADG) with a mixture of bovine and autologous bone [67]. Both groups achieved comparable regeneration, but the ADG group exhibited lower expression of TNF-α and BMP-4, indicating a more contained inflammatory response and confirming the osteoinductive and osteoconductive potential of dentin, without the need for donor site harvesting and with reduced surgical time [85,86,87].

The regenerative capacity of dentin blocks was demonstrated by Elraee et al., who compared autologous dentin blocks with autologous mandibular ramus bone blocks for horizontal ridge augmentation [59,88,89,90]. The dentin group showed a higher mean horizontal gain (3.52 mm vs. 2.24 mm) and less resorption [91,92,93]. Histological evaluation revealed 42% new bone in both groups, but a higher proportion of residual dentin (30%) in the test group, indicating slow and prolonged remodeling—advantageous for volume stability over time [59].

Ouyang et al. provided further clinical data by comparing APDDM to DBBM in an RCT [63]. After six months, the APDDM group demonstrated significantly greater gains in both width and height, with more pronounced subcrestal expansion than the DBBM group. However, after two years, APDDM sites showed greater volumetric resorption, though this was associated with reduced post-operative pain and swelling [94,95,96]. These findings highlight the biological benefits of autologous materials, despite their higher turnover rate [97].

Finally, Pohl et al. assessed the use of untreated dentin blocks and particulates for bone augmentation and intraosseous defect reconstruction [64,86]. After two years, implant survival was 96.4%, with minimal marginal bone loss (0.37 mm at one year and 0.58 mm at two years) [98,99,100]. The osteoconductive—and potentially osteoinductive—properties of the material were attributed to the preservation of intrinsic proteins, such as osteocalcin and osteonectin, as well as the presence of dentin stem cell niches [101,102,103].

While ATDGs consistently demonstrated favorable clinical and histological outcomes, these findings should be interpreted as correlations rather than proof of direct causality. Biological mechanisms are inferred but not conclusively established by the included trials.

### 4.1. Methodological Limitations and Critical Issues

Despite the encouraging clinical and histological outcomes, several recurring methodological limitations emerged from the analysis of the included studies, which constrain the generalizability of the findings and warrant cautious interpretation.

Firstly, there was marked heterogeneity in dentin preparation protocols. The type of tooth used (healthy, impacted, endodontically treated, or carious), the degree of demineralization (total, partial, or absent), and the disinfection and sterilization methods varied widely among studies—directly affecting the quality and predictability of the graft material [104,105,106].

Another major limitation was the small sample size: none of the studies included more than 40 patients, and most clinical and radiographic follow-ups did not exceed 12 months, limiting the ability to assess long-term stability.

Clinically, many studies excluded molar sites and fully edentulous patients, narrowing the application to anterior areas or partially dentate individuals. This restricts the applicability of results to more complex clinical scenarios.

A further shortcoming was the lack of subjective outcome evaluations, such as postoperative pain, patient-reported quality of life, or aesthetic and functional satisfaction—critical parameters in modern, patient-centered dentistry [107,108,109].

Lastly, the histological assessment methods varied significantly. While some studies employed advanced techniques such as immunohistochemistry or histomorphometry, others relied solely on qualitative descriptions.

Another major limitation is the marked heterogeneity of dentin preparation protocols. Differences in donor tooth type (anterior, premolar, molar, periodontally compromised), degree of demineralization (total, partial, or none), and sterilization/disinfection methods (autoclave, NaOH/ethanol, chairside disinfection) reduce comparability across studies and complicate meta-analytical synthesis. To facilitate contextualization of results, we have added Supplementary Table S1, which summarizes the exact preparation protocols reported in each included trial.

### 4.2. Clinical Implications and Future Perspectives

The use of biomaterials derived from autologous teeth presents a modern and biologically active regenerative solution, capable of combining sustainability, clinical efficacy, and treatment personalization. The ability to obtain graft material directly from the patient’s own tooth—without the need for bone harvesting from donor sites and with a low immunological risk—aligns with the principles of minimally invasive and eco-compatible dentistry [110,111,112].

From a clinical standpoint, autogenous tooth-derived grafts (ATDGs) offer several advantages: high osteoconductive and osteoinductive potential, initial volumetric stability, favorable integration with host tissue, and reduced post-operative discomfort. However, for these materials to be widely adopted, several gaps in the literature must be addressed [113,114].

Specifically, the following developments are recommended:

- Multicenter randomized clinical trials, with large sample sizes and a minimum follow-up of 24 months to assess long-term tissue stability and implant survival rates;
- Standardized preparation and sterilization protocols, including precise criteria for donor tooth selection, degrees of demineralization, and methods for endodontic canal cleaning [61,115];
- Subjective outcome evaluations, such as pain, functional recovery, patient satisfaction, and perceived aesthetic outcomes; and
- Detailed histomorphometric and biological analyses, to allow for consistent comparison between studies and to elucidate the cellular mechanisms involved in bone regeneration [116,117,118].

Another relevant gap concerns the absence of patient-reported outcomes (e.g., postoperative pain, esthetic satisfaction, quality of life). While most included studies focused on radiographic and histomorphometric metrics, none systematically assessed subjective endpoints. Future clinical trials should integrate PROMs (patient-reported outcome measures) alongside conventional outcomes, in line with the paradigm of patient-centered dentistry.

## 5. Conclusions

ATDGs represent a promising biological alternative to traditional grafting materials for ABR. Analysis of the nine clinical studies included in this review showed positive and consistent results in terms of new bone formation, ridge dimensional stability, and histologic integration, with comparable or superior performance to xenografts and conventional autologous grafts.

The main advantages of ATDGs lie in their biocompatibility, osteoconductivity, osteoinductive potential as well as the possibility of chairside use, which reduces costs and surgical trauma for the patient. However, there remain some critical issues related to standardization of preparation protocols, variability of donor material, and paucity of long-term follow-up.

In light of this evidence, it is hoped that larger-scale randomized clinical trials with uniform protocols and follow-ups longer than 24 months will be initiated to consolidate the role of ATDGs as a reliable, sustainable, and personalized option in regenerative dental surgery.

## Figures and Tables

**Table 1:** Shows the components of the PICOS criteria (population, intervention, comparison, outcome, study design), as well as their use in this evaluation [57].

**Population:** Patients undergoing alveolar bone preservation or regeneration (post-extraction or implant preparation)
**Intervention:** Use of autologous biomaterials derived from teeth (demineralised dentine, mineralised dentine, dentine blocks)
**Comparison:** Other biomaterials for grafting: autologous bone, xenografts (e.g., Bio-Oss), synthetic biomaterials Or control groups untreated/treated with PRF, collagen, or clot alone
**Outcome:** Primary: ○ New bone formation (histomorphometry) ○ Volumetric and dimensional preservation (CBCT, radiographs) Secondary: ○ Percentage of resorption ○ Residual material ○ Clinical complications/implant failures ○ Histological integration
**Study design:** Systematic review

**Table 3:** A tabular summary of the risk-of-bias assessment for the six studies, evaluated across the five domains of Rob 2.0.

Authors and Year	D1	D2	D3	D4	D5	Overall
Elfana et al. (2021) [66]	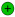	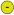	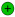	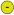	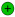	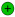
Hussain et al. (2023) [62]	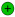	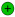	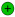	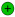	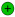	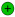
Ouyang (2024) [63]	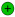	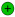	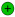	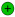	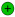	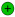
Elraee et al. (2022) [59]	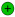	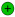	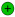	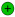	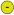	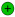
Li et al. (2018) [65]	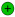	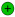	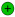	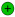	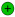	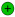
Oguić et al. (2023) [67]	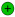	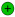	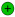	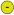	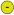	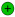

**Table 4:** A tabular summary of the risk-of-bias assessment for the three studies evaluated across the seven domains of Robins tool I.

Authors and Year	D1	D2	D3	D4	D5	D6	D7	Overall
Radoczy-Drajko et al. (2021) [60]	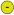	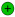	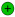	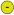	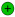	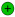	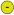	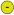
López Sacristán et al. (2024) [61]	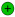	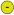	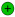	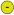	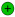	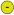	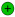	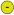
Pohl (2017) [64]	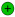	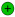	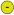	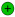	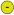	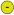	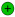	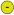

## Data Availability

No new data were created or analyzed in this study. Data sharing is not applicable to this article.

## Supplementary Materials

The following supporting information can be downloaded at: https://www.mdpi.com/article/10.3390/jfb16100367/s1.

## Abbreviations

The following abbreviations are used in this manuscript:

ADB	Autogenous Dentin Biomaterial
ADG	Autogenous Demineralized Dentin Graft
ABR	Alveolar Bone Regeneration
APDDM	Autologous Partially Demineralized Dentin Matrix
ARA	Alveolar Ridge Augmentation
ARP	Alveolar Ridge Preservation
ATDG(s)	Autologous Tooth-Derived Graft(s)
BMP(s)	Bone Morphogenetic Protein(s)
CBCT	Cone Beam Computed Tomography
DBBM	Deproteinized Bovine Bone Mineral
DDM	Demineralized Dentin Matrix
DMP-1	Dentin Matrix Protein 1
GBR	GBR Guided Bone Regeneration
HA	Hydroxyapatite
IGF-II	Insulin-like Growth Factor II
ISQ	Implant Stability Quotient
PRF	Platelet-Rich Fibrin
RCT(s)	Randomized Controlled Trial(s)
TGF-β	Transforming Growth Factor Beta

## References

[1] Ohnishi H., Fujii N., Futami T., Taguchi N., Kusakari H., Maeda T. (2000). A Histochemical Investigation of the Bone Formation Process by Guided Bone Regeneration in Rat Jaws. Effect of PTFE Membrane Application Periods on Newly Formed Bone. J. Periodontol..

[2] Nampo T., Watahiki J., Enomoto A., Taguchi T., Ono M., Nakano H., Yamamoto G., Irie T., Tachikawa T., Maki K. (2010). A New Method for Al veolar Bone Repair Using Extracted Teeth for the Graft Material. J. Periodontol..

[3] Chappuis V., Engel O., Reyes M., Shahim K., Nolte L.-P., Buser D. (2013). Ridge Alterations Post-Extraction in the Esthetic Zone. J. Dent. Res..

[4] Fan Q., Zeng H., Fan W., Wu T., Sun J., Yan Q., Shi B. (2021). Ridge Preservation of a Novel Extraction Socket Applying Bio-Oss^®^ Collagen: An Experimental Study in Dogs. J. Dent. Sci..

[5] Tan W.L., Wong T.L.T., Wong M.C.M., Lang N.P. (2012). A Systematic Review of Post-Extractional Alveolar Hard and Soft Tissue Dimensional Changes in Humans. Clin. Oral Implant. Res..

[6] Arthur A., Rychkov G., Shi S., Koblar S.A., Gronthos S. (2008). Adult Human Dental Pulp Stem Cells Differentiate toward Functionally Active Neurons under Appropriate Environmental. Cues. Stem Cells.

[7] Clark D., Rajendran Y., Paydar S., Ho S., Cox D., Ryder M., Dollard J., Kao R.T. (2018). Advanced Platelet-Rich Fibrin and Freeze-Dried Bone Allograft for Ridge Preservation: A Randomized Controlled Clinical Trial. J. Periodontol..

[8] Development of a Novel Bone Grafting Material Using Autogenous Teeth-PubMed. https://pubmed.ncbi.nlm.nih.gov/20060336/.

[9] Lin W.L., McCulloch C.A., Cho M.I. (1994). Differentiation of Periodontal Ligament Fibroblasts into Osteoblasts during Socket Healing after Tooth Extraction in the Rat. Anat. Rec..

[10] Mancini A., Laforgia A., Dipalma1 G., Inchingolo A.D., Chieppa S., Colonna V., Tartaglia F.C., Corsalini M., Palermo A., Bordea I.R. (2024). Difficulties And Perspectives Regarding Botulinum Injection And Bruxism. https://ricerca.uniba.it/handle/11586/530499.

[11] Chiapasco M., Zaniboni M., Boisco M. (2006). Augmentation Procedures for the Rehabilitation of Deficient Edentulous Ridges with Oral Implants. Clin. Oral Implant. Res..

[12] Sakkas A., Wilde F., Heufelder M., Winter K., Schramm A. (2017). Autogenous Bone Grafts in Oral Implantology-Is It Still a “Gold Standard”? A Consecutive Review of 279 Patients with 456 Clinical Procedures. Int. J. Implant. Dent..

[13] (1826). Removal of the Alveolar Processes of Both Jaws. Med. Chir. Rev..

[14] Ling Y., Chen D., Li P., Zeng X., Xu W. (2024). Repairing Alveolar Bone Defect Using Demineralized Dentin Grafts: A Meta-Analysis of Randomized Controlled Trials. BMC Oral Health.

[15] Valerio C.S., e Alves C.A., Manzi F.R. (2019). Reproducibility of Cone-Beam Computed Tomographic Measurements of Bone Plates and the Interdental Septum in the Anterior Mandible. Imaging Sci. Dent..

[16] Rapone B., Ferrara E., Qorri E., Inchingolo F., Isola G., Dongiovanni P., Tartaglia G.M., Scarano A. (2023). Research Efficacy of Gaseous Ozone Therapy as an Adjuvant to Periodontal Treatment on Oxidative Stress Mediators in Patients with Type 2 Diabetes: A Randomized Clinical Trial. BMC Oral Health.

[17] Schliephake H., Dard M., Planck H., Hierlemann H., Stern U. (2000). Alveolar Ridge Repair Using Resorbable Membranes and Autogenous Bone Particles with Simultaneous Placement of Implants: An Experimental Pilot Study in Dogs. Int. J. Oral Maxillofac. Implant..

[18] Walter C., Schmidt J.C., Rinne C.A., Mendes S., Dula K., Sculean A. (2020). Cone Beam Computed Tomography (CBCT) for Diagnosis and Treatment Planning in Periodontology: Systematic Review Update. Clin. Oral Investig..

[19] Yamada M., Egusa H. (2018). Current Bone Substitutes for Implant Dentistry. J. Prosthodont. Res..

[20] Dimitriou R., Tsiridis E., Giannoudis P.V. (2005). Current Concepts of Molecular Aspects of Bone Healing. Injury.

[21] Koga T., Minamizato T., Kawai Y., Miura K.-I., Takashi I., Nakatani Y., Sumita Y., Asahina I. (2016). Bone Regeneration Using Dentin Matrix Depends on the Degree of Demineralization and Particle Size. PLoS ONE.

[22] Kawai T., Urist M.R. (1989). Bovine Tooth-Derived Bone Morphogenetic Protein. J. Dent. Res..

[23] Almagro M.I., Roman-Blas J.A., Bellido M., Castañeda S., Cortez R., Herrero-Beaumont G. (2013). PTH [1-34] Enhances Bone Response around Titanium Implants in a Rabbit Model of Osteoporosis. Clin. Oral Implant. Res..

[24] Bessho K., Tagawa T., Murata M. (1990). Purification of Rabbit Bone Morphogenetic Protein Derived from Bone, Dentin, and Wound Tissue after Tooth Extraction. J. Oral Maxillofac. Surg..

[25] Nart J., Barallat L., Jimenez D., Mestres J., Gómez A., Carrasco M.A., Violant D., Ruíz-Magaz V. (2017). Radiographic and Histological Evaluation of Deproteinized Bovine Bone Mineral vs. Deproteinized Bovine Bone Mineral with 10% Collagen in Ridge Preservation. A Randomized Controlled Clinical Trial. Clin. Oral Implant. Res..

[26] Maiorana C., Beretta M., Salina S., Santoro F. (2005). Reduction of Autogenous Bone Graft Resorption by Means of Bio-Oss Coverage: A Prospective Study. Int. J. Periodontics Restor. Dent..

[27] Schwarz F., Sahin D., Becker K., Sader R., Becker J. (2019). Autogenous Tooth Roots for Lateral Extraction Socket Augmentation and Staged Implant Placement. A Prospective Observational Study. Clin. Oral Implant. Res..

[28] Inchingolo F., Vermesan D., Inchingolo A.D., Malcangi G., Santacroce L., Scacco S., Benagiano V., Girolamo F., Cagiano R., Caprio M. (2017). Bedsores Successfully Treated with Topical Phenytoin. Acta Biomed..

[29] Bakhshalian N., Hooshmand S., Campbell S.C., Kim J.-S., Brummel-Smith K., Arjmandi B.H. (2013). Biocompatibility and Microstructural Analysis of Osteopromotive Property of Allogenic Demineralized Dentin Matrix. Int. J. Oral Maxillofac. Implant..

[30] Ye L., MacDougall M., Zhang S., Xie Y., Zhang J., Li Z., Lu Y., Mishina Y., Feng J.Q. (2004). Deletion of Dentin Matrix Protein-1 Leads to a Partial Failure of Maturation of Predentin into Dentin, Hypomineralization, and Expanded Cavities of Pulp and Root Canal during Postnatal Tooth Development. J. Biol. Chem..

[31] Linde A. (1989). Dentin Matrix Proteins: Composition and Possible Functions in Calcification. Anat. Rec..

[32] Longerich U., Crismani A., Mayr A., Walch B., Kolk A. (2025). Development of a New Ramus Anterior Vertical Reference Line for the Evaluation of Skeletal and Dental Changes as a Decision Aid for the Treatment of Crowding in the Lower Jaw: Extraction vs. Nonextraction. J. Clin. Med..

[33] Shamsoddin E., Houshmand B., Golabgiran M. (2019). Biomaterial Selection for Bone Augmentation in Implant Dentistry: A Systematic Review. J. Adv. Pharm. Technol. Res..

[34] Jensen S.S., Terheyden H. (2009). Bone Augmentation Procedures in Localized Defects in the Alveolar Ridge: Clinical Results with Different Bone Grafts and Bone-Substitute Materials. Int. J. Oral Maxillofac. Implant..

[35] Haugen H.J., Lyngstadaas S.P., Rossi F., Perale G. (2019). Bone Grafts: Which Is the Ideal Biomaterial?. J. Clin. Periodontol..

[36] Hoeppner L.H., Secreto F., Jensen E.D., Li X., Kahler R.A., Westendorf J.J. (2009). Runx2 and Bone Morphogenic Protein 2 Regulate the Expression of an Alternative Lef1 Transcript during Osteoblast Maturation. J. Cell Physiol..

[37] Adibrad M., Shahabuei M., Sahabi M. (2009). Significance of the Width of Keratinized Mucosa on the Health Status of the Supporting Tissue around Implants Supporting Overdentures. J. Oral Implant..

[38] Le B.T., Borzabadi-Farahani A. (2014). Simultaneous Implant Placement and Bone Grafting with Particulate Mineralized Allograft in Sites with Buccal Wall Defects, a Three-Year Follow-up and Review of Literature. J. Craniomaxillofac Surg..

[39] Ritchie H.H., Ritchie D.G., Wang L.H. (1998). Six Decades of Dentinogenesis Research. Historical and Prospective Views on Phosphophoryn and Dentin Sialoprotein. Eur. J. Oral Sci..

[40] Schropp L., Wenzel A., Kostopoulos L., Karring T. (2003). Bone Healing and Soft Tissue Contour Changes Following Single-Tooth Extraction: A Clinical and Radiographic 12-Month Prospective Study. Int. J. Periodontics Restor. Dent..

[41] Reddi A.H. (1974). Bone Matrix in the Solid State: Geometric Influence on Differentiation of Fibroblasts. Adv. Biol. Med. Phys..

[42] Urist M.R., Strates B.S. (1971). Bone Morphogenetic Protein. J. Dent. Res..

[43] Ballini A., Santacroce L., Cantore S., Bottalico L., Dipalma G., Topi S., Saini R., De Vito D., Inchingolo F. (2019). Probiotics Efficacy on Oxidative Stress Values in Inflammatory Bowel Disease: A Randomized Double-Blinded Placebo-Controlled Pilot Study. Endocr. Metab. Immune Disord. Drug Targets.

[44] Morrison S.J., White P.M., Zock C., Anderson D.J. (1999). Prospective Identification, Isolation by Flow Cytometry, and in Vivo Self-Renewal of Multipotent Mammalian Neural Crest Stem Cells. Cell.

[45] Bonino F., Steffensen B., Natto Z., Hur Y., Holtzman L.P., Weber H.-P. (2018). Prospective Study of the Impact of Peri-Implant Soft Tissue Properties on Patient-Reported and Clinically Assessed Outcomes. J. Periodontol..

[46] Handschin A.E., Egermann M., Trentz O., Wanner G.A., Kock H.-J., Zünd G., Trentz O.A. (2006). Cbfa-1 (Runx-2) and Osteocalcin Expression by Human Osteoblasts in Heparin Osteoporosis in Vitro. Clin. Appl. Thromb. Hemost..

[47] Yoshida T., Vivatbutsiri P., Morriss-Kay G., Saga Y., Iseki S. (2008). Cell Lineage in Mammalian Craniofacial Mesenchyme. Mech. Dev..

[48] Fontana F., Santoro F., Maiorana C., Iezzi G., Piattelli A., Simion M. (2008). Clinical and Histologic Evaluation of Allogeneic Bone Matrix versus Autogenous Bone Chips Associated with Titanium-Reinforced e-PTFE Membrane for Vertical Ridge Augmentation: A Prospective Pilot Study. Int. J. Oral Maxillofac. Implant..

[49] Cl Clinical Application of Auto-Tooth Bone Graft Material. https://www.jkaoms.org/journal/view.html?volume=38&number=1&page=2.

[50] Araújo M.G., Lindhe J. (2005). Dimensional Ridge Alterations Following Tooth Extraction. An Experimental Study in the Dog. J. Clin. Periodontol..

[51] Al-Quisi A.F., Mohammed Aldaghir O., Al-Jumaily H.A. (2022). Comparison between Rolled and Non-rolled U-Shaped Flap in the Second Stage of Dental Implant Surgery: A Randomized Clinical Trial. Int. J. Dent..

[52] Bessho K., Tagawa T., Murata M. (1992). Comparison of Bone Matrix-Derived Bone Morphogenetic Proteins from Various Animals. J. Oral Maxillofac. Surg..

[53] Gultekin B.A., Bedeloglu E., Kose T.E., Mijiritsky E. (2016). Comparison of Bone Resorption Rates after Intraoral Block Bone and Guided Bone Regeneration Augmentation for the Reconstruction of Horizontally Deficient Maxillary Alveolar Ridges. BioMed Res. Int..

[54] Checchi V., Gasparro R., Pistilli R., Canullo L., Felice P. (2019). Clinical Classification of Bone Augmentation Procedure Failures in the Atrophic Anterior Maxillae: Esthetic Consequences and Treatment Options. BioMed Res. Int..

[55] Jeong K.-I., Kim S.-G., Kim Y.-K., Oh J.-S., Jeong M.-A., Park J.-J. (2011). Clinical Study of Graft Materials Using Autogenous Teeth in Maxillary Sinus Augmentation. Implant. Dent..

[56] Troiano G., Zhurakivska K., Lo Muzio L., Laino L., Cicciù M., Lo Russo L. (2018). Combination of Bone Graft and Resorbable Membrane for Alveolar Ridge Preservation: A Systematic Review, Meta-Analysis, and Trial Sequential Analysis. J. Periodontol..

[57] Huang X., Lin J., Demner-Fushman D. (2006). Evaluation of PICO as a Knowledge Representation for Clinical Questions. AMIA Annu. Symp. Proc..

[58] Page M.J., McKenzie J.E., Bossuyt P.M., Boutron I., Hoffmann T.C., Mulrow C.D., Shamseer L., Tetzlaff J.M., Akl E.A., Brennan S.E. (2021). The PRISMA 2020 Statement: An Updated Guideline for Reporting Systematic Reviews. BMJ.

[59] Elraee L., Abdel Gaber H.K., Elsayed H.H., Adel-Khattab D. (2022). Autogenous Dentin Block versus Bone Block for Horizontal Alveolar Ridge Augmentation and Staged Implant Placement: A Randomized Controlled Clinical Trial Including Histologic Assessment. Clin. Oral Implant. Res..

[60] Radoczy-Drajko Z., Windisch P., Svidro E., Tajti P., Molnar B., Gerber G. (2021). Clinical, Radiographical and Histological Evaluation of Alveolar Ridge Preservation with an Autogenous Tooth-Derived Particulate Graft in EDS Class 3-4 Defects. BMC Oral Health.

[61] López Sacristán H., Del Canto Pingarrón M., Alobera Gracia M.A., de Elío Oliveros J., Díaz Pedrero R., Seco-Calvo J. (2024). Use of Autologous Tooth-Derived Material as a Graft in the Post-Extraction Socket. Split-Mouth Study with Radiological and Histological Analysis. BMC Oral Health.

[62] Hussain A.A., Al-Quisi A.F., Abdulkareem A.A. (2023). Efficacy of Autogenous Dentin Biomaterial on Alveolar Ridge Preservation: A Randomized Controlled Clinical Trial. BioMed Res. Int..

[63] Ouyang L., Li J., Dong Y., Li J., Jin F., Luo Y., Wang R., Wang S. (2024). Comparison of Clinical Efficacy between Autologous Partially Demineralized Dentin Matrix and Deproteinized Bovine Bone Mineral for Bone Augmentation in Orthodontic Patients with Alveolar Bone Deficiency: A Randomized Controlled Clinical Trial. BMC Oral Health.

[64] Pohl V., Pohl S., Sulzbacher I., Fuerhauser R., Mailath-Pokorny G., Haas R. (2017). Alveolar Ridge Augmentation Using Dystopic Autogenous Tooth: 2-Year Results of an Open Prospective Study. Int. J. Oral Maxillofac. Implant..

[65] Li P., Zhu H., Huang D. (2018). Autogenous DDM versus Bio-Oss Granules in GBR for Immediate Implantation in Periodontal Postextraction Sites: A Prospective Clinical Study. Clin. Implant. Dent. Relat. Res..

[66] Elfana A., El-Kholy S., Saleh H.A., Fawzy El-Sayed K. (2021). Alveolar Ridge Preservation Using Autogenous Whole-Tooth versus Demineralized Dentin Grafts: A Randomized Controlled Clinical Trial. Clin. Oral Implant. Res..

[67] Oguić M., Čandrlić M., Tomas M., Vidaković B., Blašković M., Jerbić Radetić A.T., Zoričić Cvek S., Kuiš D., Cvijanović Peloza O. (2023). Osteogenic Potential of Autologous Dentin Graft Compared with Bovine Xenograft Mixed with Autologous Bone in the Esthetic Zone: Radiographic, Histologic and Immunohistochemical Evaluation. Int. J. Mol. Sci..

[68] Amer O., Shemais N., Fawzy El-Sayed K., Saleh H.A., Darhous M. (2025). Does Injectable Platelet-Rich Fibrin Combined With Autogenous Demineralized Dentine Enhance Alveolar Ridge Preservation? A Randomized Controlled Trial. Clin. Oral Implant. Res..

[69] Cardaropoli G., Araújo M., Lindhe J. (2003). Dynamics of Bone Tissue Formation in Tooth Extraction Sites. An experimental study in dogs. J. Clin. Periodontol..

[70] Avila-Ortiz G., Chambrone L., Vignoletti F. (2019). Effect of Alveolar Ridge Preservation Interventions Following Tooth Extraction: A Systematic Review and Meta-Analysis. J. Clin. Periodontol..

[71] Frigério P.B., Gomes-Ferreira P.H.S., de Souza Batista F.R., Moura J., Rangel Garcia Júnior I., Botticelli D., Lisboa-Filho P.N., Okamoto R. (2021). Effect of Topical PTH 1-34 Functionalized to Biogran^®^ in the Process of Alveolar Repair in Rats Submitted to Orchiectomy. Materials.

[72] Ramanauskaite A., Sahin D., Sader R., Becker J., Schwarz F. (2019). Efficacy of Autogenous Teeth for the Reconstruction of Alveolar Ridge Deficiencies: A Systematic Review. Clin. Oral Investig..

[73] Schwarz F., Hazar D., Becker K., Sader R., Becker J. (2018). Efficacy of Autogenous Tooth Roots for Lateral Alveolar Ridge Augmentation and Staged Implant Placement. A prospective controlled clinical study. J. Clin. Periodontol..

[74] Cho H.-L., Lee J.-K., Um H.-S., Chang B.-S. (2010). Esthetic Evaluation of Maxillary Single-Tooth Implants in the Esthetic Zone. J. Periodontal. Implant. Sci..

[75] Signorini L., Ballini A., Arrigoni R., De Leonardis F., Saini R., Cantore S., De Vito D., Coscia M.F., Dipalma G., Santacroce L. (2021). Evaluation of a Nutraceutical Product with Probiotics, Vitamin D, Plus Banaba Leaf Extracts (*Lagerstroemia speciosa*) in Glycemic Control. Endocr. Metab. Immune Disord. Drug Targets.

[76] Chisci G., Hatia A., Chisci E., Chisci D., Gennaro P., Gabriele G. (2023). Socket Preservation after Tooth Extraction: Particulate Autologous Bone vs. Deproteinized Bovine Bone. Bioengineering.

[77] Bassir S.H., Alhareky M., Wangsrimongkol B., Jia Y., Karimbux N. (2018). Systematic Review and Meta-Analysis of Hard Tissue Outcomes of Alveolar Ridge Preservation. Int. J. Oral Maxillofac. Implant..

[78] Jafer M.A., Salem R.M., Hakami F.B., Ageeli R.E., Alhazmi T.A., Bhandi S., Patil S. (2022). Techniques for Extraction Socket Regeneration for Alveolar Ridge Preservation. J. Contemp. Dent. Pract..

[79] Hazballa D., Inchingolo A.D., Inchingolo A.M., Malcangi G., Santacroce L., Minetti E., Di Venere D., Limongelli L., Bordea I.R., Scarano A. (2021). The Effectiveness of Autologous Demineralized Tooth Graft for the Bone Ridge Preservation: A Systematic Review of the Literature. J. Biol. Regul. Homeost. Agents.

[80] Berglundh T., Armitage G., Araujo M.G., Avila-Ortiz G., Blanco J., Camargo P.M., Chen S., Cochran D., Derks J., Figuero E. (2018). Peri-Implant Diseases and Conditions: Consensus Report of Workgroup 4 of the 2017 World Workshop on the Classification of Periodontal and Peri-Implant Diseases and Conditions. J. Clin. Periodontol..

[81] Cochran D.L., Jones A., Heijl L., Mellonig J.T., Schoolfield J., King G.N. (2003). Periodontal Regeneration with a Combination of Enamel Matrix Proteins and Autogenous Bone Grafting. J. Periodontol..

[82] Zhao L., Zhan Y., Hu W., Xu T., Wei Y., Zhen M., Wang C. (2016). Evaluation with Different Measuring Methods for the Alveolar Bone Change of Ridge Preservation in Molar Sites. Beijing Da Xue Xue Bao Yi Xue Ban.

[83] Ronda M., Rebaudi A., Torelli L., Stacchi C. (2014). Expanded vs. Dense Polytetrafluoroethylene Membranes in Vertical Ridge Augmentation around Dental Implants: A Prospective Randomized Controlled Clinical Trial. Clin. Oral Implant. Res..

[84] Schwarz F., Golubovic V., Becker K., Mihatovic I. (2016). Extracted Tooth Roots Used for Lateral Alveolar Ridge Augmentation: A Proof-of-Concept Study. J. Clin. Periodontol..

[85] Feng J.Q., Luan X., Wallace J., Jing D., Ohshima T., Kulkarni A.B., D’Souza R.N., Kozak C.A., MacDougall M. (1998). Genomic Organization, Chromosomal Mapping, and Promoter Analysis of the Mouse Dentin Sialophosphoprotein (Dspp) Gene, Which Codes for Both Dentin Sialoprotein and Dentin Phosphoprotein. J. Biol. Chem..

[86] Pohl V., Fürhauser L., Haas R., Pohl S. (2020). Gingival Recession Behavior with Immediate Implant Placement in the Anterior Maxilla with Buccal Dehiscence without Additional Augmentation-a Pilot Study. Clin. Oral Investig..

[87] Maréchal M. (2002). Guided bone augmentation in edentulous areas. Ned. Tijdschr. Tandheelkd..

[88] Avila-Ortiz G., Gonzalez-Martin O., Couso-Queiruga E., Wang H.-L. (2020). The Peri-Implant Phenotype. J. Periodontol..

[89] Laforgia A., Inchingolo A.D., Piras F., Colonna V., Giorgio R.V., Carone C., Rapone B., Malcangi G., Inchingolo A.M., Inchingolo F. (2024). Therapeutic Strategies and Genetic Implications for Periodontal Disease Management: A Systematic Review. Int. J. Mol. Sci..

[90] Kuboki Y., Hashimoto F., Ishibashi K. (1988). Time-Dependent Changes of Collagen Crosslinks in the Socket after Tooth Extraction in Rabbits. J. Dent. Res..

[91] Gottlow J., Nyman S., Karring T., Lindhe J. (1984). New Attachment Formation as the Result of Controlled Tissue Regeneration. J. Clin. Periodontol..

[92] Buser D., Ruskin J., Higginbottom F., Hardwick R., Dahlin C., Schenk R.K. (1995). Osseointegration of Titanium Implants in Bone Regenerated in Membrane-Protected Defects: A Histologic Study in the Canine Mandible. Int. J. Oral Maxillofac. Implant..

[93] Kuroshima S., Al-Salihi Z., Yamashita J. (2013). Parathyroid Hormone Related to Bone Regeneration in Grafted and Nongrafted Tooth Extraction Sockets in Rats. Implant. Dent..

[94] Benic G.I., Thoma D.S., Jung R.E., Sanz-Martin I., Unger S., Cantalapiedra A., Hämmerle C.H.F. (2017). Guided Bone Regeneration with Particulate vs. Block Xenogenic Bone Substitutes: A Pilot Cone Beam Computed Tomographic Investigation. Clin. Oral Implant. Res..

[95] Retzepi M., Donos N. (2010). Guided Bone Regeneration: Biological Principle and Therapeutic Applications. Clin. Oral Implant. Res..

[96] Inchingolo F., Inchingolo A.M., Latini G., de Ruvo E., Campanelli M., Palermo A., Fabbro M.D., Blasio M.D., Inchingolo A.D., Dipalma G. (2024). Guided Bone Regeneration: CGF and PRF Combined With Various Types of Scaffolds-A Systematic Review. Int. J. Dent..

[97] Inchingolo A.D., Patano A., Coloccia G., Ceci S., Inchingolo A.M., Marinelli G., Malcangi G., Di Pede C., Garibaldi M., Ciocia A.M. (2022). Treatment of Class III Malocclusion and Anterior Crossbite with Aligners: A Case Report. Medicina.

[98] Botticelli D., Berglundh T., Lindhe J. (2004). Hard-Tissue Alterations Following Immediate Implant Placement in Extraction Sites. J. Clin. Periodontol..

[99] Claflin R.S. (1936). Healing of Disturbed and Undisturbed Extraction Wounds. J. Am. Dent. Assoc. 1922.

[100] Beck T.M., Mealey B.L. (2010). Histologic Analysis of Healing after Tooth Extraction with Ridge Preservation Using Mineralized Human Bone Allograft. J. Periodontol..

[101] Berglundh T., Abrahamsson I., Welander M., Lang N.P., Lindhe J. (2007). Morphogenesis of the Peri-Implant Mucosa: An Experimental Study in Dogs. Clin. Oral Implant. Res..

[102] Juodzbalys G., Stumbras A., Goyushov S., Duruel O., Tözüm T.F. (2019). Morphological Classification of Extraction Sockets and Clinical Decision Tree for Socket Preservation/Augmentation after Tooth Extraction: A Systematic Review. J. Oral Maxillofac. Res..

[103] Rich A. (2009). Most Cited: Number 4. The Healing of Extraction Wounds: An Experimental Study Based on Microscopic and Radiographic Investigations. N. Z. Dent. J..

[104] Chen H., Fu T., Ma Y., Wu X., Li X., Li X., Shen J., Wang H. (2017). Intermittent Administration of Parathyroid Hormone Ameliorated Alveolar Bone Loss in Experimental Periodontitis in Streptozotocin-Induced Diabetic Rats. Arch. Oral Biol..

[105] Esposito M., Grusovin M.G., Felice P., Karatzopoulos G., Worthington H.V., Coulthard P. (2009). Interventions for Replacing Missing Teeth: Horizontal and Vertical Bone Augmentation Techniques for Dental Implant Treatment. Cochrane Database Syst. Rev..

[106] Adams R.J. (2022). Is There Clinical Evidence to Support Alveolar Ridge Preservation over Extraction Alone? A Review of Recent Literature and Case Reports of Late Graft Failure. Br. Dent. J..

[107] Hashemi S., Tabatabaei S., Baghaei K., Fathi A., Atash R. (2024). Long-Term Clinical Outcomes of Single Crowns or Short Fixed Partial Dentures Supported by Short (≤6 Mm) Dental Implants: A Systematic Review. Eur. J. Dent..

[108] Keestra J.A.J., Barry O., de Jong L., Wahl G. (2016). Long-Term Effects of Vertical Bone Augmentation: A Systematic Review. J. Appl. Oral Sci..

[109] Simion M., Fontana F., Rasperini G., Maiorana C. (2004). Long-Term Evaluation of Osseointegrated Implants Placed in Sites Augmented with Sinus Floor Elevation Associated with Vertical Ridge Augmentation: A Retrospective Study of 38 Consecutive Implants with 1- to 7-Year Follow-Up. Int. J. Periodontics Restor. Dent..

[110] Xu H., Shimizu Y., Ooya K. (2005). Histomorphometric Study of the Stability of Newly Formed Bone after Elevation of the Floor of the Maxillary Sinus. Br. J. Oral Maxillofac. Surg..

[111] Parvini P., Schliephake C., Al-Maawi S., Schwarz K., Sader R., Ghanaati S., Schwarz F. (2020). Histomorphometrical Assessment of Vertical Alveolar Ridge Augmentation Using Extracted Tooth Roots in the Canine. Clin. Oral Investig..

[112] Urban I.A., Lozada J.L., Jovanovic S.A., Nagy K. (2013). Horizontal Guided Bone Regeneration in the Posterior Maxilla Using Recombinant Human Platelet-Derived Growth Factor: A Case Report. Int. J. Periodontics Restor. Dent..

[113] Salata L.A., Hatton P.V., Devlin A.J., Craig G.T., Brook I.M. (2001). In Vitro and in Vivo Evaluation of E-PTFE and Alkali-Cellulose Membranes for Guided Bone Regeneration. Clin. Oral Implant. Res..

[114] Schwarz F., Schmucker A., Becker J. (2016). Initial Case Report of an Extracted Tooth Root Used for Lateral Alveolar Ridge Augmentation. J. Clin. Periodontol..

[115] Dipalma G., Inchingolo A.M., Lauria P., Marotti P., Chieppa S., Venere D.D., Palermo A., Corsalini M., Inchingolo F., Inchingolo A.D. (2025). Unilateral Agenesis of the Upper Permanent Lateral Incisors in Growing Patients: Gap Closure or Gap Opening? A Systematic Review. Int. Dent. J..

[116] Stevens A., Zuliani T., Olejnik C., LeRoy H., Obriot H., Kerr-Conte J., Formstecher P., Bailliez Y., Polakowska R.R. (2008). Human Dental Pulp Stem Cells Differentiate into Neural Crest-Derived Melanocytes and Have Label-Retaining and Sphere-Forming Abilities. Stem Cells Dev..

[117] Morelli T., Neiva R., Wang H.-L. (2009). Human Histology of Allogeneic Block Grafts for Alveolar Ridge Augmentation: Case Report. Int. J. Periodontics Restor. Dent..

[118] Nkenke E., Hahn M., Weinzierl K., Radespiel-Tröger M., Neukam F.W., Engelke K. (2003). Implant Stability and Histomorphometry: A Correlation Study in Human Cadavers Using Stepped Cylinder Implants. Clin. Oral Implant. Res..

