# Supplementary material for: Autologous Tooth-Derived Biomaterials in Alveolar Bone Regeneration: A Systematic Review of Clinical Outcomes and Histological Evidence
Source: J Funct Biomater. 2025 Oct 1;16(10):367. doi: 10.3390/jfb16100367 (PMC12565049; doi:10.3390/jfb16100367)
Supplement: Supplementary file 1 [file jfb-16-00367-s001.zip › jfb-3807664-supplementary Table S1.pdf]

**Study**	**Tooth type**	**Demineralization**	**Sterilization/Disinfection**	**Notes**
**Elraee et al. (2022) [59]**	Mandibular teeth	Demineralized dentin blocks	Autoclave	Compared to autologous bone blocks
**Elfana et al. (2021) [66]**	Single-rooted teeth	Demineralized dentin	Autoclave	Compared to whole-tooth graft
**Hussain et al. (2023) [62]**	Anterior teeth	No demineralization	Chairside grinding + disinfection	Autogenous dentin biomaterial
**Li et al. (2018) [65]**	Periodontal compromised teeth	Demineralized dentin matrix	Proprietary system	Compared to Bio-Oss®
**López Sacristán et al. (2024) [61]**	Premolars/molars	No demineralization	Grinding + disinfection	Fresh dentin graft
**Oguić et al. (2023) [67]**	Esthetic zone teeth	Demineralized dentin	Chemical disinfection	Compared to bovine + autologous bone
**Ouyang et al. (2024) [63]**	Orthodontic premolars	Partially demineralized dentin	Proprietary APDDM sterilization	Compared to DBBM
**Pohl (2017) [64]**	Mixed teeth	No demineralization	Chairside disinfection	Untreated dentin blocks/particulate
**Radoczy-Drajko et al. (2021) [60]**	Mixed teeth	Partially demineralized dentin	Chemical disinfection (NaOH, ethanol)	Bonmaker® protocol
